# Systemic barriers and opportunities for implementing school-based social–emotional learning interventions in low-income and conflict-affected settings

**DOI:** 10.3389/fpsyg.2023.1011039

**Published:** 2023-03-06

**Authors:** Dana Charles McCoy, Emily C. Hanno

**Affiliations:** Human Development, Learning, and Teaching, Harvard Graduate School of Education, Cambridge, MA, United States

**Keywords:** social–emotional learning, low-and middle-income countries, violence, conflict, implementation

## Abstract

Children living in low-income and conflict-affected settings face unique systemic risk factors that shape their social, emotional, and mental well-being. However, little is known about how these and other systemic factors may impede or support the delivery of social–emotional learning (SEL) interventions in these contexts. In this article, we draw from our experience delivering and evaluating a classroom-based SEL curriculum in Rio de Janeiro, Brazil to surface systemic barriers and opportunities for implementing SEL interventions in low-income, conflict-affected settings. Specifically, we identify (1) culture, (2) timing, and (3) government support and stability as factors underlying SEL program demand, dosage, quality, and effectiveness. We provide recommendations for improving implementation of SEL programs in low-income and conflict-affected contexts, including the importance of building pro-active partnerships, using qualitative research, and investing in adaptation to both understand and address systemic barriers.

## 1. Introduction

Nearly 90 percent of children live in a low-or middle-income country (LMIC; World Bank, 2019) and one in six lives in a conflict zone (Kamøy et al., 2021). Children growing up in low-income and conflict-affected settings face unique risks that may jeopardize their social–emotional well-being, including exposure to trauma and reduced access to protective resources (Black et al., 2017; Murphy et al., 2017). Indeed, 10 to 20 percent of children and adolescents living in LMICs are affected by a mental health problem, with many cases thought to be preventable (Kieling et al., 2011).

School-based social–emotional learning (SEL) supports – including direct instruction in SEL strategies and/or teacher training in positive behavior management and stress reduction – have been shown to meaningfully improve children’s social–emotional wellbeing and mental health (Durlak et al., 2011; Wigelsworth et al., 2016; Taylor et al., 2017; Blewitt et al., 2018). Importantly, this evidence largely comes from high-income countries, with substantially less known about school-based SEL programming in non-Western, low-income, and/or violence-afflicted settings (Barry et al., 2013), which are characterized by a complex system of risks, resources, and cultural imperatives that likely also affect program implementation.

In this Perspective article, we draw from our experiences evaluating a school-based SEL intervention in Rio de Janeiro, Brazil to describe possible barriers and opportunities for implementing SEL programming in low-income, conflict-affected settings. Notably, whereas much of the literature on intervention implementation has focused on the individual-, school-, or community-level factors that shape SEL program dosage, fidelity, and reach (e.g., teacher characteristics, school climate; Durlak and DuPre, 2008; Durlak, 2016), here we emphasize broader macro-and exo-systemic considerations that may either promote or interfere with SEL implementation in low-income, conflict-affected contexts. In doing so, our goal is to identify potential paths forward for supporting children’s social–emotional wellbeing in these under-represented contexts. To support our arguments, we incorporate evidence from our own work alongside findings from a small but growing set of published SEL program evaluations in LMICs and high-violence settings (e.g., Ştefan and Miclea, 2013; Huang et al., 2017; Baker-Henningham and Walker, 2018; Bilir Seyhan et al., 2019; Torrente et al., 2019; Aber et al., 2021; Tubbs Dolan et al., 2022). Nevertheless, we acknowledge that our points are largely speculative and need to be confirmed with future research.

## 2. Overview of the Programa Compasso evaluation

This article draws from our experiences implementing Programa Compasso (“Compass Program”) within a randomized control trial conducted in 90 primary schools across Rio de Janeiro, Brazil in 2017. A middle-income country, Brazil is characterized by robust social services for families and high economic inequality, violence, and instability. In particular, Brazil’s homicide rate is about five times the global average (27 vs. 5 per 100,000 people, respectively), ranking it among the most violent countries in the world (UN Office on Drugs and Crime, 2021).

Programa Compasso is a universal, school-wide SEL curriculum that includes 22 weekly, 50 min lessons delivered to students by classroom teachers. These scripted lessons provide direct instruction in emotion knowledge, self-regulation, executive function, empathy, and social problem solving strategies that is reinforced by activities, games, and materials. Lessons were adapted from the United States-based Second Step program by a Brazilian NGO, Instituto Vila Educação. After piloting the Programa Compasso curriculum in 17 schools in São Paulo in 2015, Instituto Vila Educação made slight modifications to the lessons and expanded the program to include student workbooks to reinforce content at home, a parent engagement component, and teacher trainings focused on improving implementation.

The evaluation of Programa Compasso was funded by a Brazilian education-focused foundation and included 90 schools that were randomized within matched pairs at the beginning of 2017 to either an intervention or a business-as-usual control condition. A total of 3,018 students from 90 third-and 90 fifth-grade classrooms took part in the evaluation, which included teachers’ reports of student behavior problems and group-administered direct assessments of student executive function and emotion knowledge as outcomes. Results of the 2017 evaluation showed no average impacts of the Programa Compasso intervention after 1 year on the five outcomes tested. We did, however, observe small, positive program impacts (*d* = 0.15 *SD*s) on students’ labeling of emotional expressions and inhibitory control in communities characterized by below-average levels of violence. Although budgetary limitations and local data collection restrictions prevented us from collecting detailed implementation data to formally contextualize these impacts, findings from a voluntary end-of-year teacher survey suggested that responding treatment teachers delivered an average of just 13 of the 22 intended lessons. Furthermore, consistent with prior SEL evaluations in conflict-affected settings (e.g., Tubbs Dolan et al., 2022), student attendance was generally quite low during the intervention period, suggesting limited take-up. For additional details of the intervention, study design, and results, see McCoy et al. (2021).

## 3. Barriers and opportunities for ensuring SEL program implementation

### 3.1. Culture

Based on our experience with Programa Compasso, perhaps the most salient systemic influence on the implementation of SEL programming in low-income, conflict-affected settings is *culture*. Cultural values prioritizing SEL in Brazil provided an opportunity for our study to take place by fomenting initial demand for SEL services (Durlak and DuPre, 2008). Brazilians generally adhere to horizontal collectivist values, emphasizing group well-being and prosocial behavior alongside individual equality (Carlo et al., 2007; Martinez et al., 2020). Although Brazilians report that the learning of these values begins at home, they also believe that the education system is key to equipping children –particularly from low-income backgrounds –with skills to get along with others and endure the hardships of everyday life (Dessen and Torres, 2002). Indeed, Brazil’s national learning standards –codified in the Base Nacional Comum Curricular (BNCC) –explicitly emphasize SEL skills as outcomes of public education, including responsibility and citizenship, empathy and cooperation, self-knowledge and self-care, and critical and creative thinking (Movimento Pela Base Nacional Comum, 2018). In our experience, these collective values around (1) the importance of SEL and (2) the central role that schools play in its socialization generated the community appetite that allowed our work to be funded, implemented, and taken-up in Rio. Had we attempted this work in a different cultural context (e.g., areas of sub-Saharan Africa where parental demand prioritizes schools’ academic rigor; Bidwell et al., 2014; Wolf, 2020), we speculate that gaining community buy-in would have been more difficult.

Culture also positively *and* negatively shaped the structure and implementation of Programa Compasso itself. Prior meta-analysis quantifying the “cross-cultural transportability” of SEL programming suggests that interventions implemented outside the countries in which they were developed tend to be less effective for improving certain outcomes than those implemented in their country of origin (Wigelsworth et al., 2016). Accordingly, considering cultural relevance is important for optimizing the success of school-based SEL programming in LMICs and conflict-affected settings. To maximize the cultural appropriateness of Programa Compasso, Instituto Vila Educação added a student workbook and parent meetings to the original US-based Second Step curriculum to more explicitly reflect the centrality of the family system in Brazil (Carlo et al., 2007) and teachers’ beliefs that the program would not work without investments from parents. The core lessons from Second Step were also modified, but mostly in minor ways (e.g., replacing references to skiing with football/soccer) to avoid tampering with the program’s “active ingredients” (Durlak, 2016).

Although many teachers praised the final intervention content and structure as “relevant” and “productive,” some reported that the program remained “decontextualized from [their] own reality.” One educator, for example, questioned the importance of teaching students to individually regulate (read: control) their emotions, saying instead that Brazilian children should “embrace” emotions, sharing and co-regulating them with peers and caregivers. Teachers also sometimes pushed back against the scripted nature of the lessons, instead pursuing *ad hoc* approaches to teaching the SEL topic of the week. We speculate several different opportunities could have mitigated these challenges. For example, more substantive –or “deep structure” (Ahluwalia et al., 1999) –adaptations to Programa Compasso’s lesson content could have improved cultural alignment. Alternatively, helping teachers to understand how existing lessons could be used to support context-specific goals (e.g., co-regulation) may have improved their motivation to implement the program as-is, with greater fidelity.

Finally, our findings highlight the importance of viewing culture not just as a stable, broad-scale influence, but also as a force that shapes SEL program implementation and effectiveness more locally (Vélez-Agosto et al., 2017). Our evaluation showed that Programa Compasso improved child outcomes within neighborhoods characterized by lower-than-average levels of violence; however, in higher-violence communities, the program showed no impacts. Independent of the broader cultural values of Brazil as a whole, these findings could reflect more localized variability in the socialization practices used in safer versus less safe environments. Indeed, most SEL programs –including Programa Compasso –take an “approach” orientation to teaching conflict resolution, encouraging children to stop, think, and discuss their feelings with others. Although these mainstream strategies seem to have been modestly effective in low-violence neighborhoods, they may have contradicted the avoidant strategies often taught to protect children’s physical safety in conflict-affected settings (e.g., to quickly disengage from conflict, run away, etc.; Kliewer et al., 2006), limiting their applicability, take-up, and effectiveness in Rio’s more dangerous communities. Once again, these results reinforce the importance of aligning SEL programmatic strategies with cultural values. Importantly, however, they also encourage taking a narrower, more localized view of culture to avoid fallacies regarding cultural homogeneity (e.g., within all LMICs, within Brazil, etc.).

### 3.2. Timing

A second noteworthy factor affecting SEL implementation in low-income, conflict-affected settings is *timing*. Around the start of our study in 2017, the federal Ministry of Education ratified the BNCC, increasing political appetite for curricular approaches targeting SEL-related learning standards. Simultaneously, Brazil was experiencing widespread gang violence and police shootouts amidst an economic recession and several highly publicized government corruption scandals. In particular, Rio experienced a 26 percent surge in community crime (Fonseca and Alper, 2018), forcing schools to close for so-called “violence days” during 99 of the first 107 school days in 2017 (de Oliveira, 2017). Even when children were attending school, educators reported concerns with students’ social–emotional wellbeing, noting that children “are here, but their head is always outside” (Londoño, 2017). Finally, a simultaneous move from part-to full-day schooling for many primary schools in Rio effectively doubled the “supply” of instructional hours available to meet the demand for SEL programming inspired by the BNCC and rise in community violence. Collectively, these “opportunities” opened doors for us to implement Programa Compasso in Rio. Nevertheless, the stress these same factors placed on educators may have also negatively affected their capacity to deliver the intervention with sufficient dose or fidelity. For example, more than 60 percent of teachers in our sample said that violence affects their school at least “a little,” and approximately 70 percent said that either they or their students had trouble getting to school because of violence.

Overall, these findings suggest that the timing of broader political, cultural, or societal events can present as both opportunities *and* barriers for program implementation. In low-income and conflict-affected settings, destabilizing events like the ones we observed in Rio (e.g., bursts of violence, policy shifts) are especially common, and often result in calls for supporting the social–emotional needs of children. Nevertheless, as we observed in our study, the stress and instability created by these events may also limit the bandwidth of individuals tasked with delivering SEL supports. Such tensions were also observed globally amidst the COVID-19 pandemic, as widespread teacher burnout coincided with broader demand for and availability of SEL services (Reimers et al., 2020; Gultom et al., 2022). Taking advantage of the opportunities to affect change presented by these broader social shocks, while also compensating for the additional stress that they place on program implementers, could be one path forward for successful implementation of SEL programming in LMICs and high-violence areas.

Such shocks may also open opportunities for creative SEL solutions that either supplement or replace traditional school-based approaches. Community-or technology-based programming (e.g., parent groups, apps) may be particularly useful for reaching children when they cannot attend school safely. Virtual SEL programs have been especially popular since the onset of the COVID-19 pandemic (Katzman and Stanton, 2020). In 2021, for example, our team worked with Rio’s Ministry of Education to deliver lessons on stress management remotely via television. Although these non-school-based approaches to SEL hold promise for addressing access gaps during times of crisis, evidence regarding their effectiveness is still emerging.

### 3.3. Government support and stability

A third systemic factor that we observed to underpin SEL implementation is *government support and stability*. In many LMIC and conflict-affected settings, government officials (e.g., staff from Ministries of Education) influence not only whether an SEL program is taken up, but whether it is sustained and how it is implemented. Government turnover is a major issue globally, and especially in contexts characterized by instability and public mistrust. In Brazil, it is common for new governments to abolish or severely restructure programs established by their predecessors. In the case of Programa Compasso, the 2016 municipal elections in Rio led to staffing changes in the Ministry of Education that coincided with the start of our study. Although we were fortunate to receive permission from the new government to continue our research, the initial enthusiasm we received from the Ministry of Education was tempered as new officials took office and focused on their own agendas. We speculate that these changes ultimately affected implementation, with new government staff providing less oversight, guidance, and encouragement for schools to deliver Programa Compasso than their predecessors.

Even in non-election years, inconsistencies in government oversight and support can affect SEL implementation by breeding mistrust from program implementers. Some teachers in our study voiced resistance to Programa Compasso solely because it was mandated by officials who they perceived as unfamiliar with and unsupportive of their day-to-day work. Despite the shift to full-day teaching, teachers reported being over-worked, having limited time, and needing social–emotional services for *themselves* before they could support their students. Studies have shown similar patterns of government mistrust in Brazil, with many teachers pushing for increased autonomy regarding resource allocation and curricular planning (Lennert da Silva and Mølstad, 2020).

Despite these barriers, durable partnerships between NGOs and public officials whose jobs are not tied to a particular political party or election result (i.e., “comissionados” in Brazil) could help to sustain implementation in the face of government instability. Although we lacked such partnerships in Rio, we have seen in other areas of Brazil that collaborations focused outwardly on advocacy, awareness-raising, and empowerment may be particularly effective. For example, efforts led by a coalition of foundations to educate political candidates and the public in Ceará, Brazil about the importance of the early years have helped to ensure the popularity –and longevity –of early childhood programs in the state (Fundação Maria Cecilia Souto Vidigal, 2021). Furthermore, internal dialogs focused on building trust and equality between government staff and program implementers could help to overcome the issues of mistrust that we observed in our study. In particular, such partnerships can support implementers’ (e.g., teachers’) understanding of the value of a given SEL program while also improving their capacity to make improvements aligned with government goals. Indeed, experimental evidence from Brazil has shown that shifting decision-making authority from governments to teachers can reduce teacher turn-over and improve student learning and social–emotional outcomes (Piza et al., 2020).

## 4. Recommendations and paths forward

Overcoming the challenges highlighted above (along with additional challenges common to low-income, high-violence contexts but not explicitly considered here) requires creative solutions. Beyond directly addressing systemic barriers (e.g., through broad-scale violence reduction efforts), there are several steps that researchers and practitioners can take *before* implementing SEL programs that may improve delivery and uptake in LMICs and conflict-affected settings. First, investments in durable, trusting partnerships with decisionmakers and advocates (e.g., government officials, NGO staff, funders) are critical for gauging initial demand for SEL programming, for promoting a sense of co-ownership that maintains this demand and associated supports over time, and for overseeing implementation (Durlak and DuPre, 2008). Such partnerships take time to build and sustain, and must be based on mutual trust, responsiveness, patience, and flexibility (Aber et al., 2021). Critically, teachers and other implementers should be included in these partnerships to maximize their buy-in as active contributors to the programming that they are ultimately expected to deliver, and to ensure that efforts to oversee implementation are not perceived as reducing their autonomy. Second, understanding the cultural and political appetite for SEL programming, as well as whether the timing is right for proceeding, is a must. Implementing SEL interventions in unwelcoming contexts is likely to be a Sisyphean task. Conducting qualitative research with a variety of parties (e.g., government officials, teachers, and families) can identify potential systemic roadblocks like those described above, along with more localized opportunities and barriers within communities or school systems (Tinajero et al., 2016). Working with teachers to sensitize them to the benefits of SEL programs –both for their students *and* their own well-being –is also critical. Third, adaptation of imported programmatic content and structures is needed to maximize political and cultural relevance. As noted above, adaptation should ensure alignment not only with broad-scale cultural norms (e.g., collectivist values), but also more localized, community-and school-specific priorities. Once again, this adaptation should occur in partnership with program implementers and beneficiaries, as well as researchers familiar with local SEL program best practices (Durlak, 2016).

## 5. Discussion

Demand for SEL programming in low-income, conflict-affect settings is high. Nevertheless, the same systemic factors that increase children’s risk for social–emotional challenges in these settings also shape SEL service selection, delivery, and take-up. Our experiences implementing and evaluating a classroom-based SEL program for primary students in Rio de Janeiro, Brazil highlighted (1) culture, (2) timing, and (3) government support and stability as key systemic factors underlying SEL program demand, implementation, and effectiveness in LMICs and conflict-affected settings. Understanding and addressing these factors through partnerships with government, NGO, school, community, and family collaborators is critical for optimizing the potential of SEL programming *before* implementation begins.

BOX 1 Key steps for successful implementation of SEL programs in low-income, conflict-affected settings.

1. Invest in partnerships

- Build relationships between interested parties to share information, equalize power dynamics, and develop co-ownership.
- Ensure involvement of multiple parties, including government officials (ideally whose positions are not tied to a particular political party or election result), NGO staff, funders, researchers, community leaders, program implementers (e.g., school leaders, teachers), and program beneficiaries (e.g., students, families).
- Allow plenty of time to build partnerships *before* making key decisions about program implementation.

2. Understand the context

- Conduct qualitative research to gauge initial demand for SEL programming in the particular setting, as well as barriers and opportunities for ongoing implementation.
- Pay particular attention to systemic barriers and opportunities related to (1) culture, (2) timing, and (3) government support and stability.
- Involve multiple interested parties to understand contextual needs at multiple “levels,” ranging from broader government systems to specific community/school priorities.

3. Adapt as needed

- Adjust program content and structures to address key cultural factors prior to implementation. Ensure that cultural adaptations are attuned to both broad-scale cultural norms and localized priorities (e.g., of a given type of community or school).
- Invest in high-quality, autonomy-focused training that (1) educates program implementers (e.g., teachers) regarding the value of SEL programming for their students *and* themselves, and (2) guides them regarding how content should and should not be further adapted to meet their needs without compromising core program ingredients.
- Involve multiple experts in the adaptation process, including local researchers, implementers, and beneficiaries.
- Consider the timing of broader social events (e.g., elections, policy shifts, outbreaks of violence) when deciding when and how to implement. Address any specific barriers emerging from these events before proceeding.
- Do not be afraid to pivot or cancel implementation entirely if conditions are not right.

## Data availability statement

The original contributions presented in the study are included in the article/supplementary material, further inquiries can be directed to the corresponding author.

## Author contributions

DM conceived the manuscript structure, devised the argument, and drafted the manuscript. EH contributed to the manuscript structure and argument, and provided critical edits to the manuscript text. All authors contributed to the article and approved the submitted version.

## Funding

This work was supported by a series of grants from the Harvard Lemann Brazil Research Fund. The funder had no role in the design, execution, analysis, or reporting of the research reported in this article.

## Conflict of interest

The authors declare that the research was conducted in the absence of any commercial or financial relationships that could be construed as a potential conflict of interest.

## Publisher’s note

All claims expressed in this article are solely those of the authors and do not necessarily represent those of their affiliated organizations, or those of the publisher, the editors and the reviewers. Any product that may be evaluated in this article, or claim that may be made by its manufacturer, is not guaranteed or endorsed by the publisher.
